# Human Macrophage Responses to Clinical Isolates from the *Mycobacterium tuberculosis* Complex Discriminate between Ancient and Modern Lineages

**DOI:** 10.1371/journal.ppat.1001307

**Published:** 2011-03-03

**Authors:** Damien Portevin, Sébastien Gagneux, Iñaki Comas, Douglas Young

**Affiliations:** 1 Mycobacterial Research Division, MRC National Institute for Medical Research, London, United Kingdom; 2 Department of Medical Parasitology and Infection Biology, Swiss Tropical and Public Health Institute, Basel, Switzerland; 3 University of Basel, Basel, Switzerland; New York Medical College, United States of America

## Abstract

The aim of the present study was to determine whether there is a correlation between phylogenetic relationship and inflammatory response amongst a panel of clinical isolates representative of the global diversity of the human *Mycobacterium tuberculosis* Complex (MTBC). Measurement of cytokines from infected human peripheral blood monocyte-derived macrophages revealed a wide variation in the response to different strains. The same pattern of high or low response to individual strains was observed for different pro-inflammatory cytokines and chemokines, and was conserved across multiple human donors. Although each major phylogenetic lineage of MTBC included strains inducing a range of cytokine responses, we found that overall inflammatory phenotypes differed significantly across lineages. In particular, comparison of evolutionarily modern lineages demonstrated a significant skewing towards lower early inflammatory response. The differential response to ancient and modern lineages observed using GM-CSF derived macrophages was also observed in autologous monocyte-derived dendritic cells and murine bone marrow-derived macrophages, but not in human unfractionated peripheral blood mononuclear cells. We hypothesize that the reduced immune responses to modern lineages contribute to more rapid disease progression and transmission, which might be a selective advantage in the context of expanding human populations. In addition to the lineage effects, the large strain-to-strain variation in innate immune responses elicited by MTBC will need to be considered in tuberculosis vaccine development.

## Author Summary

*Mycobacterium tuberculosis* is a long-standing human pathogen spread by aerosol transmission between individuals interacting in close social groups. It can be anticipated that the evolution of *M. tuberculosis* will parallel the evolution of human societies, and the phylogeny as determined by whole genome sequencing of clinical isolates is indeed consistent with emergence of the pathogen with modern humans in Africa and its subsequent dissemination along routes of human migration and trade. The present study was designed to test the hypothesis that the genetic diversity of *M. tuberculosis* isolates would be reflected in a corresponding diversity in their biological properties. In particular, we explored the interaction of different isolates with the innate immune system, which plays important contrasting roles in initial resistance to infection and in disease transmission. We observed a difference in the innate immune response when we compared isolates belonging to “modern” lineages that have evolved amongst high-density populations in regions of recent massive demographic expansion, with isolates belonging to “ancient” lineages selected in older low-density human populations. Our results provide insights into host-pathogen co-evolution and into fundamental mechanisms underlying the pathogenesis of *M. tuberculosis*.

## Introduction

High-throughput sequence analysis has allowed reconstruction of the evolution of human *Mycobacterium tuberculosis* Complex (MTBC), differentiating the bacteria into six main phylogenetic lineages [1], [2]. Three lineages, including two whose members are known as *M. africanum* [3], which branched off from a common ancestor at an early stage of evolution, are referred to as evolutionarily “ancient” lineages; three separate evolutionarily “modern” lineages diverged at a later time point [1]. It is proposed that the branches reflect the history of human migration out of Africa, with the current geographic distribution of the different lineages being determined by the expansion and migration of their corresponding host populations.

This phylogeny provides a rational framework to assess whether the genotypic diversity of MTBC is associated with diversity in biological phenotype [4], [5]. Several studies suggest that this may be the case [5], [6]. A comparison of pulmonary TB with TB meningitis in Vietnam demonstrated that strains belonging to the modern Euro-American lineage were significantly less likely to cause extra-pulmonary disease [7]. In contrast, the modern Beijing/W lineage was found significantly associated with extra-thoracic TB in comparison with non-Beijing/W lineages, though no difference was found when looking at other read-outs of virulence such as the amount of cavitation [8]. A recent study in Madagascar found that infection with an ancient lineage induced a significantly higher immune response, measured as interferon-γ production by peripheral blood T cells [9]. Finally, a series of studies, including the report from Madagascar, have described a reduced immune response to members of the “Beijing family” (part of the modern East Asian Lineage 2), and its association with rapid progression to severe disease in humans and experimental animals [9], [10], [11], [12], [13], [14].

Two studies that analyzed individual isolates involved in TB outbreaks concluded that a low inflammatory response was linked to increased virulence [10], [15]. The rationale is that a reduction in innate immune recognition will result in a delay in engagement of the adaptive response, providing the pathogen with a significant advantage during the early stage of infection. The low inflammatory phenotype of *M. tuberculosis* HN878, a member of the Beijing family implicated in an outbreak in Texas, was reversed by disruption of the gene encoding an enzyme required for biosynthesis of a phenolic glycolipid molecule (PGL) [11]. However, it was shown later that the production of PGL was variable across strains from the Beijing/W lineage [16]. Moreover, the role of this particular glycolipid in the virulence of HN878 could not be reproduced by restoring its production in the genetic background of another modern strain belonging to Lineage 4, highlighting a rather confusing inter- and intra-lineage diversity in the molecular mechanisms of *M. tuberculosis* pathogenicity. In contrast, the low inflammatory phenotype of *M. tuberculosis* CAS, responsible for an outbreak in Leicester, was linked to a chromosomal deletion and could be reversed by restoration of the functional gene [15]. Several other studies have described differences in the inflammatory response induced by different isolates of *M. tuberculosis* [12], [13], [17], [18], [19].

In the present study, we used a collection of strains covering the global genetic diversity of MTBC to test the hypothesis that inflammatory phenotype would be linked to genotype.

## Results

### Human *Mycobacterium tuberculosis* Complex (MTBC) isolates differ in their induction of pro-inflammatory cytokines

**Figure 1:**
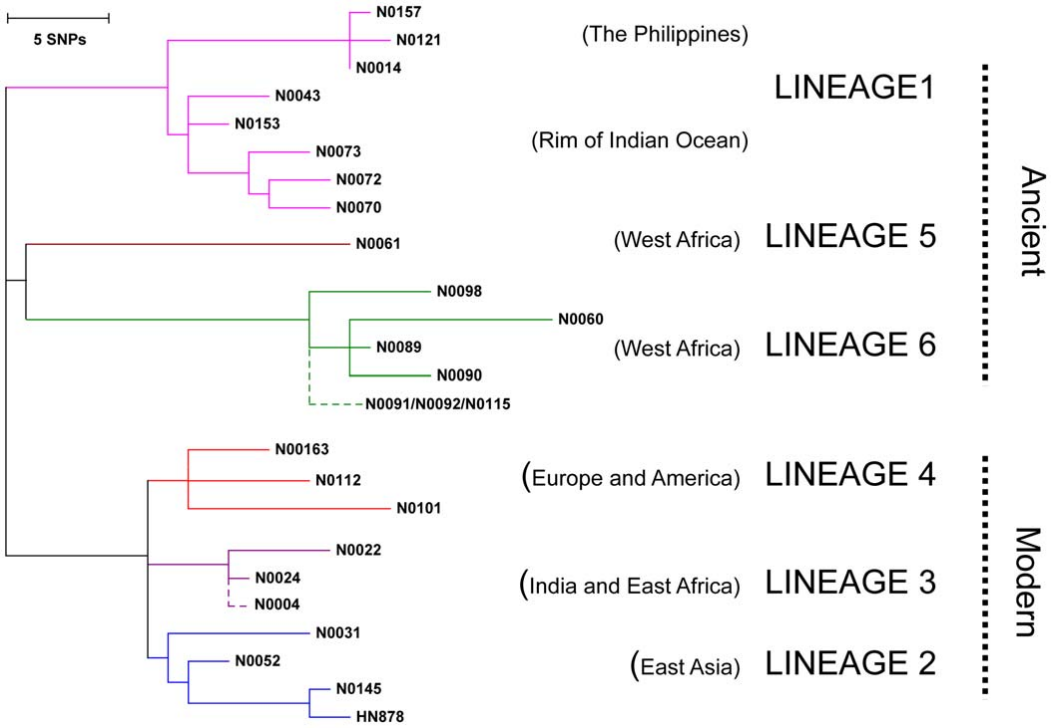
Selection of MTBC isolates representative of global genetic diversity. Phylogenetic tree of the 26 strains used for the study based on a concatenate alignment of 89 genes previously described [1]. One strain belonging to Lineage 3 and three strains to Lineage 6 were not present in the original study and are depicted with dashed lines branching from the node representing the most common recent ancestor of the respective lineage. The color code highlights the six lineages of *M. tuberculosis* complex (MTBC) strains affecting humans. These lineages were clustered in two groups according to the occurrence of the TbD1 genomic deletion which discriminates “ancient” from “modern” lineages [1], [22].

**Figure 2:**
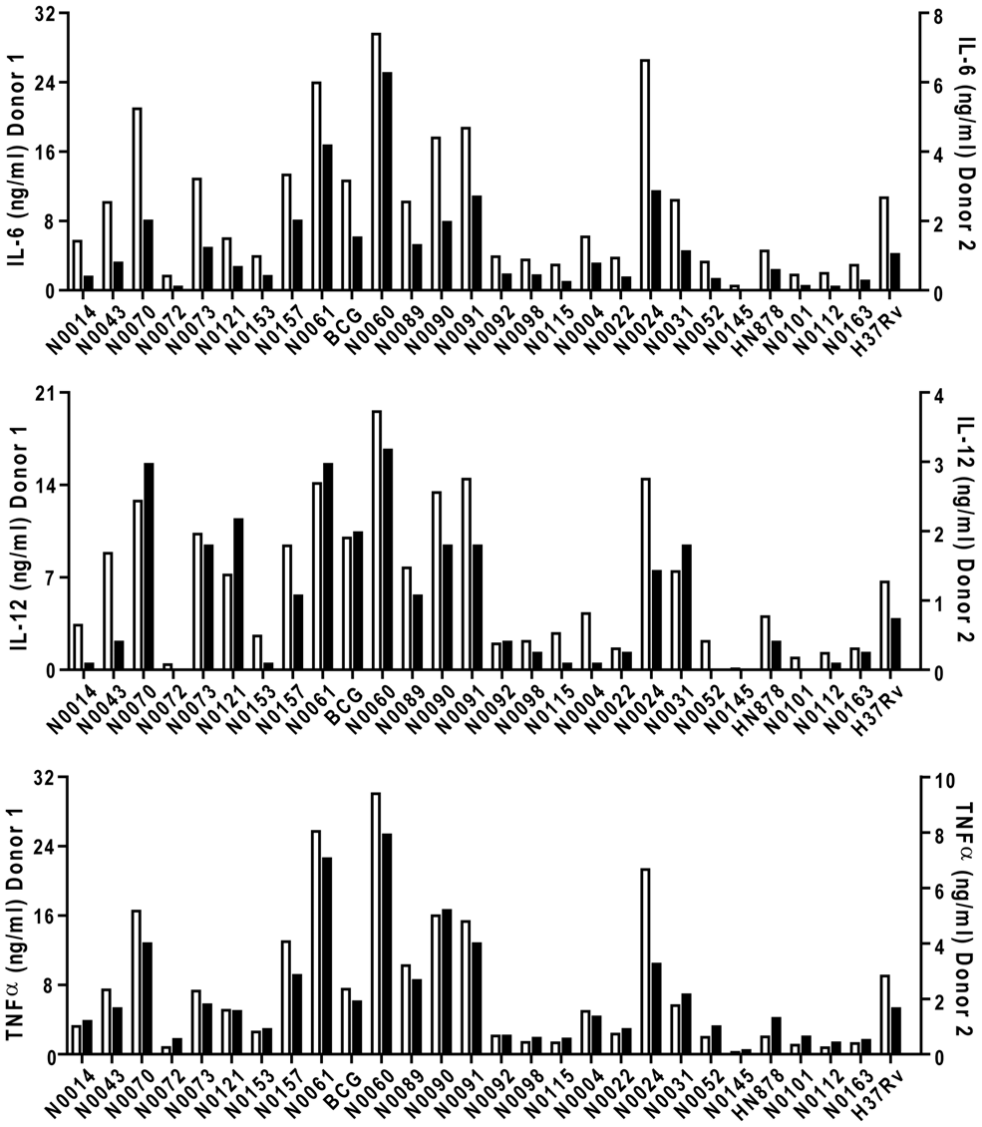
*M. tuberculosis* complex clinical isolates vary widely in their induction of pro-inflammatory cytokines. Graphic representation of the cytokine response from GM-CSF monocyte-derived macrophages of two different donors after infection with the panel of MTBC strains. IL-6, IL-12p40/p70 and TNFα were measured by ELISA from filtered supernatants 24 h post-infection (MOI 1∶1). The inflammatory response differs widely between the different strains. Cytokine responses from each donor were plotted on different scales to highlight the conservation of the trend between donors.

To test for a link between genotype and inflammatory phenotype, we selected 26 isolates representative of the global diversity of human MTBC from a well-characterized clinical strain collection [1], [4], [20] (Figure 1) plus two laboratory adapted strains as references (*M. tuberculosis* H37Rv and *M. bovis* BCG Pasteur) and measured their ability to induce production of inflammatory cytokines by human GM-CSF monocyte-derived macrophages (T1-MDMs) [21]. Figure 2 shows cytokine levels from culture supernatants harvested 24 hours after infection with each of the strains for two human donors, and highlights three important observations.

**Figure 3:**
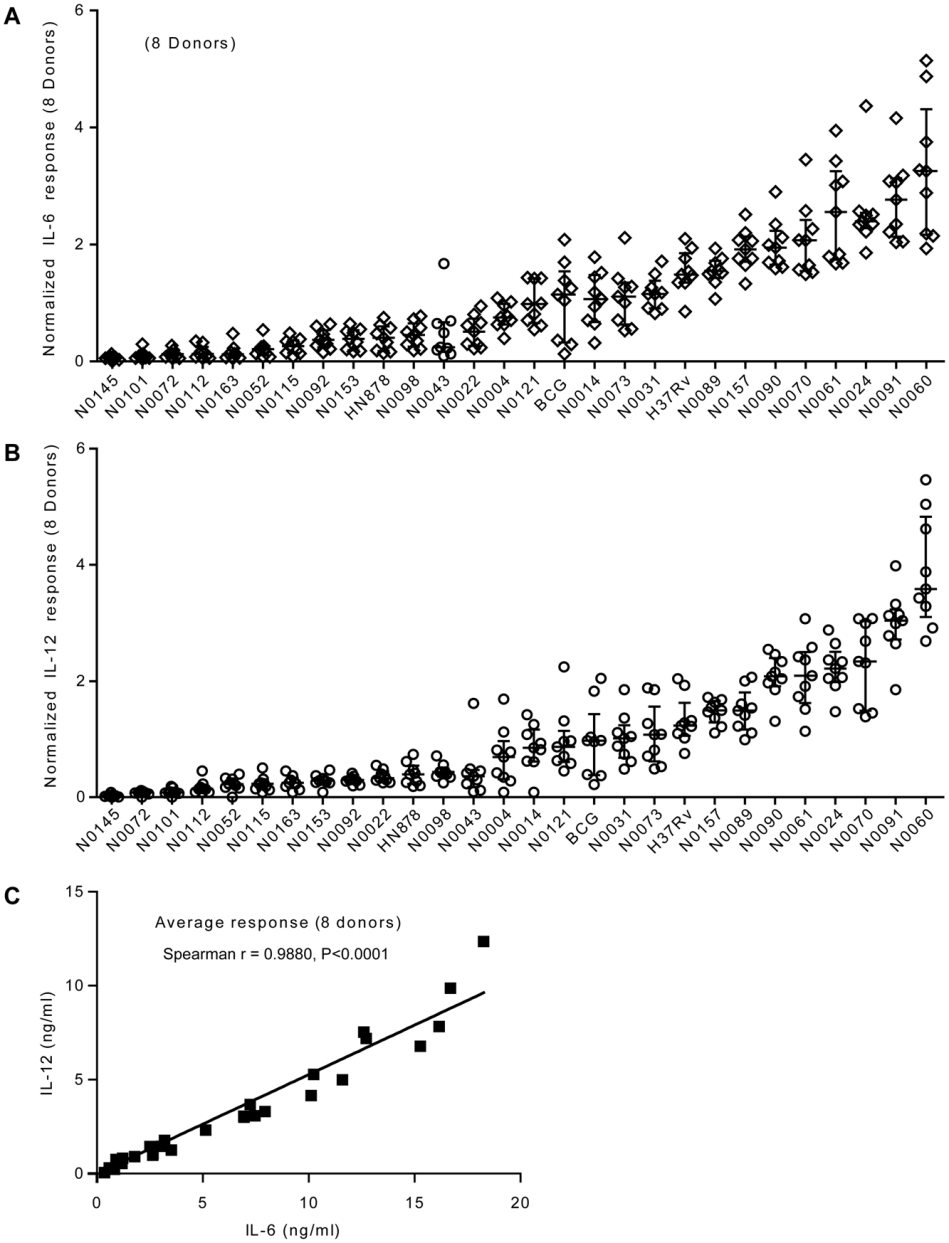
Strain-related hierarchy in pro-inflammatory response is maintained across multiple human donors. **A**) Scatter plot representation of IL-6 and **B**) IL-12p40/p70 macrophage responses from eight different individual donors. Cytokine responses from each individual donor to a particular strain were normalized with respect to the median response of the donor towards the panel of strains. The response to each strain was ranked according to the average response across the eight donors. The results show that the hierarchy of IL-6 and IL-12p40/p70 response is maintained across independent donors. **C**) Linear regression analysis shows significant correlation between IL-6 and IL-12p40/p70 macrophage responses to the different strains used in the study. Correlation coefficient and p-value result from non-parametric correlation test (Spearman) are indicated on the graph.

First, there were clear differences in the level of pro-inflammatory cytokines produced by a single donor in response to different strains; ranging from a few hundred picograms to several nanograms. Second, although the absolute amount of cytokine varies between individual donors (results in Figure 2 are plotted against two separate y-axes), the relative hierarchy of low and high responses is the same in the two donors. This is further illustrated in Figure 3, summarizing results from eight donors, with strains ranked according to their ability to induce a cytokine response after median normalization of the dataset. In addition to robust consistency across different human donors, a similar hierarchy was observed when the same strains were used to stimulate bone-marrow-derived macrophages from Balb/c mice (Figure S1). A third observation is that the different pro-inflammatory cytokines – IL-6, IL-12p40/p70 and TNFα – all showed a similar pattern. Again, this is particularly clear when analyzed across the panel of eight donors, showing a highly significant correlation between production of IL-6 and IL-12p40/p70 for example (Figure 3C; Spearman rank correlation coefficient  =  0.988, *P*<0.001).

### Innate immune responses to *M. tuberculosis* complex strains correlate with the phylogeny and genetic diversity data

**Figure 4:**
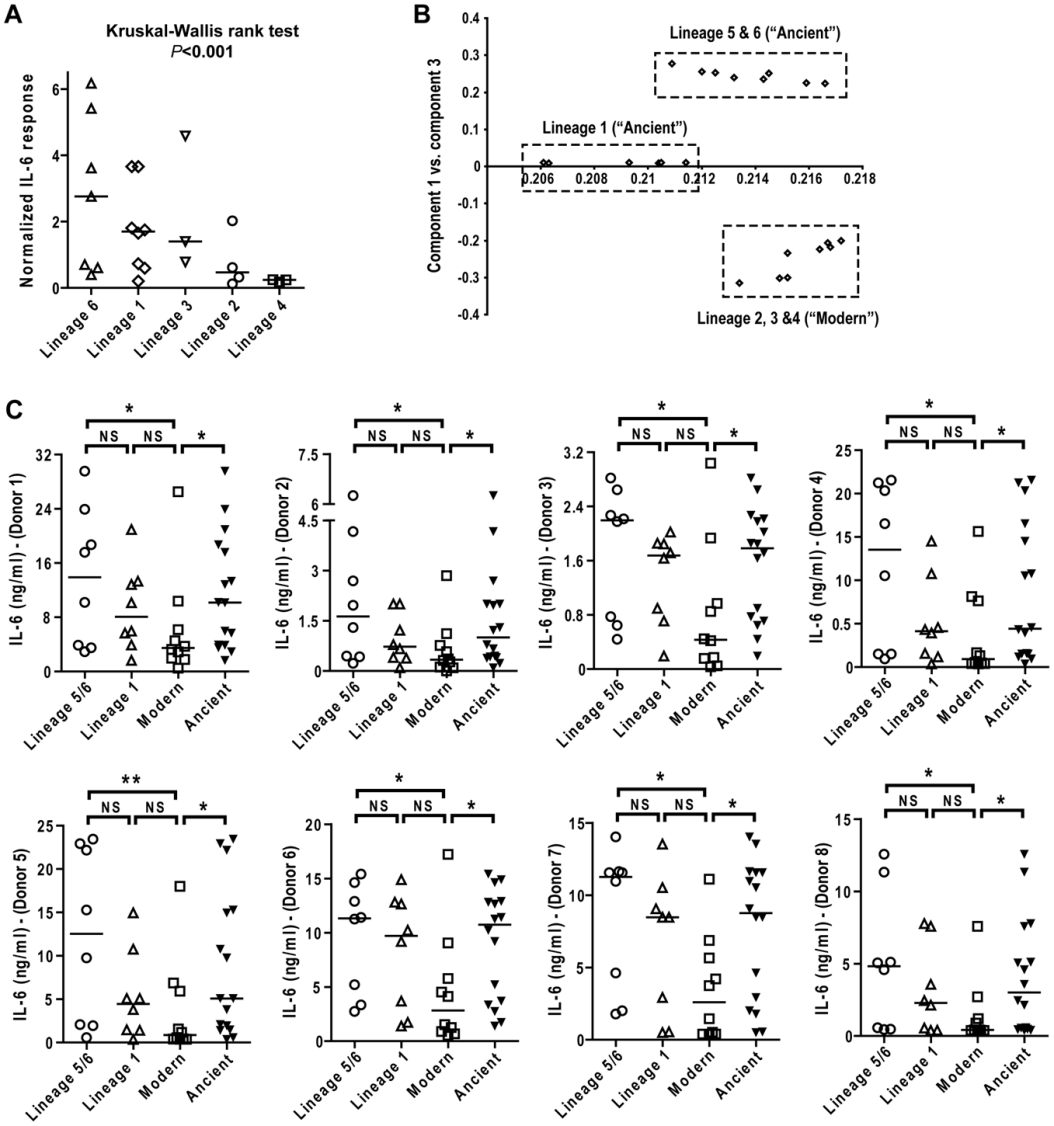
IL-6 production varies with genetic clustering of MTBC lineages. A) Median normalized levels of cytokine production by human monocyte-derived macrophages from eight independent donors were averaged for scatter plot representation and clustered according to strain lineage. Non-parametric Kruskal-Wallis test revealed a significant effect of lineage variable in cytokine production (*P*<0.001). B) PCA plot (PC1 versus PC3) of the strains used in this study based on the polymorphisms found between them in Hershberg et al. 2008 highlights three main groups clearly separating the three modern lineages from Lineage 1, and from the two *M. africanum* lineages (5 and 6). C) IL-6 response from T1-MDMs from eight donors infected by the panel of MTBC strains indicates that Lineages 5+6 and Lineage 1 induce more IL-6 than the modern lineages. (Mann-Whitney U test, * *P*<0.05, ** *P*<0.01).

We next compared the pro-inflammatory responses across the main MTBC lineages. Combining results from the eight individual donors after median normalization revealed higher heterogeneity in IL-6 production in response to Lineage 5, 6 and 1 (Figure 4A). However, there was overall a statistically significant effect of lineage on the cytokine levels observed (Kruskal-Wallis rank test, *P*<0.001). To look for higher order grouping of the lineages we performed a principal component analysis (PCA) using the genetic variation among the strains. PCA distinguished three major groups: strains belonging to Lineages 5+6 (*M. africanum*), strains belonging to Lineage 1, and strains belonging to the Lineages 2+3+4 (these three lineages have been referred to as evolutionarily “modern” based on previous work [1], [22]) (Figure 4B). These three major groupings were consistent with the most recent genome-based MTBC phylogeny published earlier this year [2] and shows that the “modern” lineages are more genetically homogenous than “ancient” lineages.

**Figure 5:**
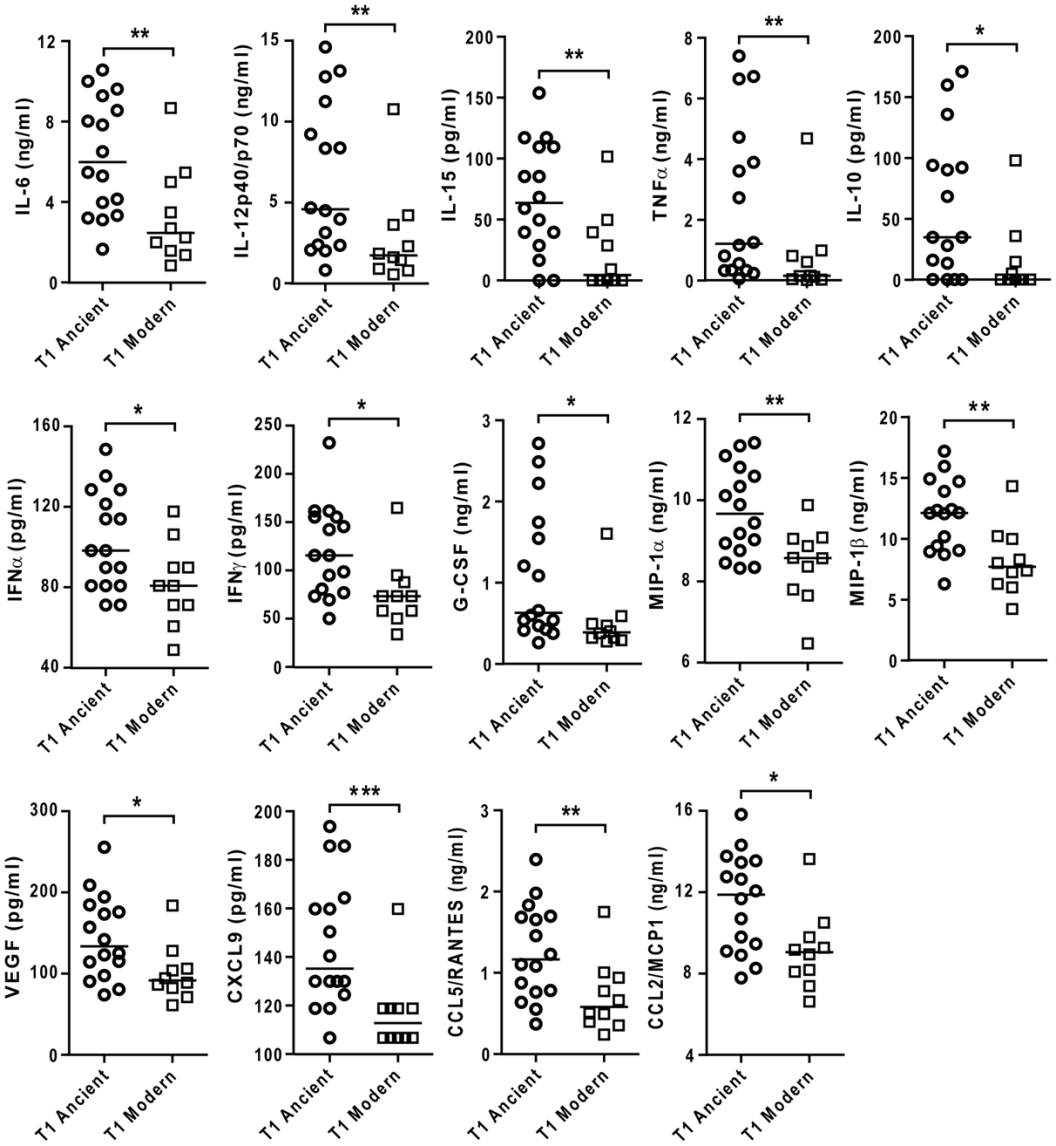
Strains from the modern lineages of *M. tuberculosis* complex induce lower levels of pro-inflammatory cytokines and chemokines. Supernatants of infected macrophages 24 h post-infection from 3 independent donors were pooled and analyzed using multiplex technology and results were plotted for each individual cytokine/chemokine. The results showed statistically significant lower responses by macrophages infected with strains belonging to the modern lineage (Mann-Whitney U test, * *P*<0.05, ** *P*<0.01).

Comparing the level of cytokine induction across these three groups showed that the modern group of strains consistently induced a lower IL-6 response when compared to Lineage 5+6 or Lineage 1 (Figure 4C), though Lineage 1 had an intermediate phenotype reflecting again the higher heterogeneity of the ancient group when compared to the modern strains. Even so, combining the lineages according to the ancient/modern grouping revealed an overall difference in inflammatory phenotype, with strains from the modern lineages always eliciting significantly lower levels of IL-6 (Figure 4C) and other cytokines and chemokines such as IL-12p40/p70, TNFα, IL-15, MIP-1α, CCL5 and others (Figure 5). Therefore for the rest of the study we decided to keep the "ancient"/"modern" dichotomy to highlight the different behavior of the later group when compared with the other lineages. However, relevant figures splitting the data in three groups ("*M. africanum*", Lineage 1 and "Modern") are made available as supplementary material (Figure S2 A-E).

As a further test of the reproducibility of the differential inflammatory response, experiments were repeated using a second batch of the same strains of MTBC separately cultured and quantified. While a few individual isolates showed evidence of batch variation in inflammatory phenotype, the overall pattern of a lower response to the modern lineage was maintained as shown by a statistically significant correlation test (Spearman correlation test, *P*<0.05) (Figure S3).

**Figure 6:**
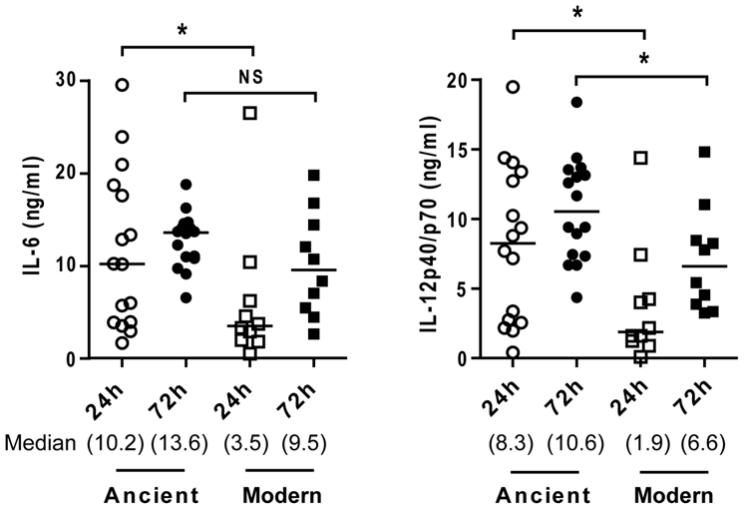
Lower cytokines production during infection with modern strains is associated with a delayed inflammatory response. Scatter plot representation of macrophage cytokine responses to ancient versus modern strains at 24 h and 72 h post-infection from a single donor. Supernatants from infected macrophages were collected at 24 h and 72 h and analyzed by ELISA for IL-6 and IL-12p40/p70 content. While the trend towards higher cytokine production from macrophages infected by strains belonging to the ancient lineage is maintained, convergence of cytokine levels at the later time point (particularly in the case of IL-6) suggests that lower responses to the modern lineage may reflect a delay rather than complete inhibition of the inflammatory response (Mann-Whitney U test, * *P*<0.05, NS non significant).

There was a differential increase in the production of pro-inflammatory cytokines as the infection progressed, and the difference between ancient and modern lineages observed at the 24-hour time point had markedly diminished by 72 hours (Figure 6; Mann-Whitney U test, *P*<0.05). Thus, the reduced response to the modern strains is due to a delay in the kinetics, rather than a complete inhibition of the immune response.

### Dependence of cytokine profile on monocyte differentiation

**Figure 7:**
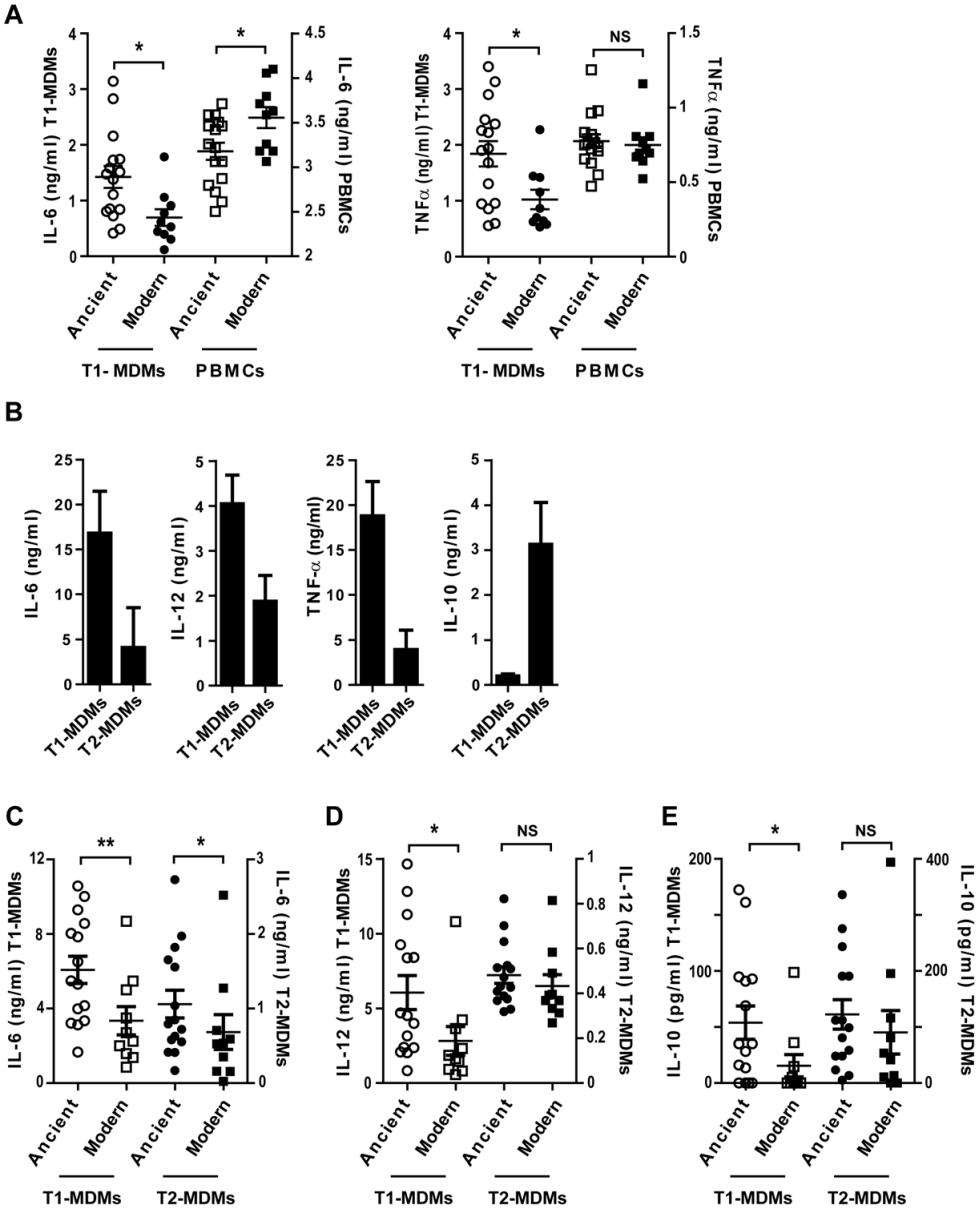
The inflammatory response to ancient and modern lineages differs when infecting autologous PBMCs or anti-inflammatory Type 2 macrophages. A) Scatter plot representation of macrophage (circles) versus autologous PBMCs (squares) cytokine responses to ancient (open) versus modern (filled) groups of strains shows that the pro-inflammatory phenotype was reversed when infecting PBMCs instead of GM-CSF derived macrophages from the same donor and looking at IL-6 production but not TNFα. B) Macrophages from three independent donors were derived from monocytes either in the presence of recombinant human GM-CSF or M-CSF to generate Type 1 macrophages (T1-MDMs) and Type 2 macrophages (T2-MDMs) respectively. Results are presented as histograms of cytokine production from LPS-stimulated T1-MDMs and T2-MDMs showing increased pro-inflammatory potential of T1-MDMs, revealed by higher levels of IL-6, IL-12p40/p70 and TNFα produced 24 h post-stimulation, in contrast to T2-MDMs which produced higher levels of anti-inflammatory IL-10. C) IL-6 D) IL-12p40/p70 E) IL-10 averaged levels from autologous T1-MDMs versus T2-MDMs (single donor) infected by the various strains of MTBC clustered into ancient and modern lineages. The higher pro-inflammatory phenotype associated with the ancient lineages is only observed for IL-6 when infecting T2-MDMs but not for IL-12p40/p70 neither for the production of anti-inflammatory IL-10 (Mann-Whitney U test, * *P*<0.05, ** *P*<0.01, NS non significant).

The differential response pattern was not observed in infection experiments using unfractionated peripheral blood mononuclear cell (PBMC) preparations in place of differentiated monocytes. In these experiments, we observed a lower amount of TNFα but with no significant difference between strains from the ancient and modern lineages, and a statistically significant higher amount of IL-6 in comparison to T1-MDMs (Figure 7A; Mann-Whitney U test, *P*<0.05).

Replacement of GM-CSF by M-CSF during monocyte differentiation generated a “Type 2” macrophage population characterized by a lower level of production of pro-inflammatory cytokines, along with increased expression of IL-10 [21]. Figure 7B illustrates the differences between Type 1 (T1-MDMs) and Type 2 (T2-MDMs) polarization in response to LPS stimulation. Comparative flow cytometry analysis is shown as supplementary material (Figure S4). Infection of T2-MDMs with the different strains of MTBC resulted in lower production of IL-6 and IL-12p40/p70 as compared to T1-MDMs, with a significant difference between lineages only for IL-6 (Figure 7C, D). In contrast to T1-MDMs, a consistent IL-10 response was induced during infection of the T2-MDMs. The IL-10 response was also variable between isolates, but no significant difference was observed when comparing ancient and modern lineages (Figure 7E).

**Figure 8:**
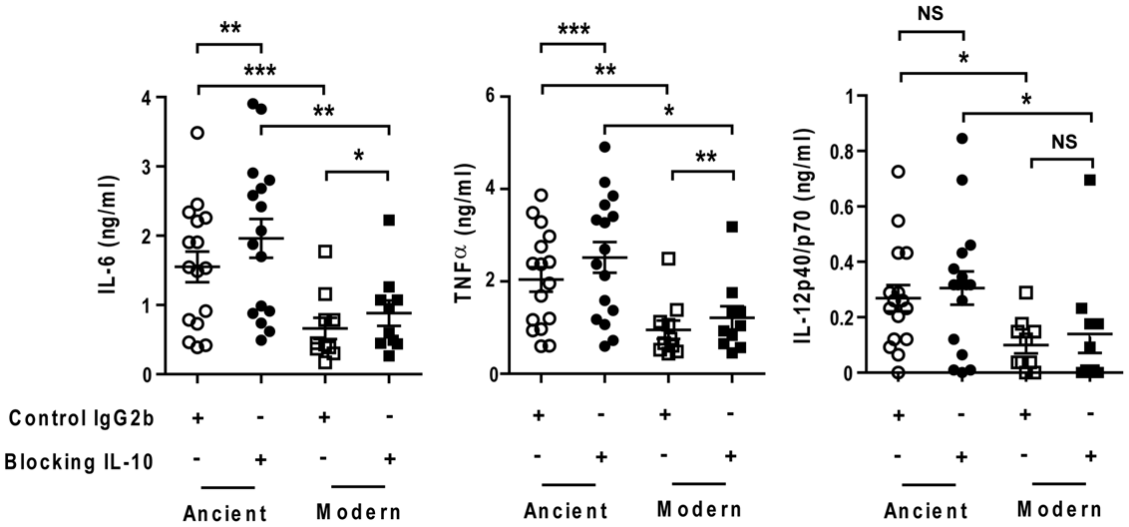
The delayed inflammatory response of modern lineage is not due to IL-10. Scatter plot representation of Type 1 macrophage cytokine response from a single donor 24 h post-infection towards ancient (circles) versus modern (squares) groups of strains in the presence (filled) or absence (open) of anti-IL-10 blocking antibodies. IL-10 blockage resulted in a significant increase in the production of IL-6 and TNFα independent of lineage. The delayed inflammatory response to strains from modern lineages is not due to IL-10 regulatory activity (Paired t-test for intra-group comparison and Mann-Whitney U test for inter-group comparison, * *P*<0.05, ** *P*<0.01 and *** *P*<0.001).

IL-10 is an anti-inflammatory cytokine that has been shown to act as an auto-regulatory inhibitor of pro-inflammatory cytokine production by human monocytes [23], [24]. Notably, IL-10 production has been associated with the anti-inflammatory phenotype of a recent outbreak strain belonging to the modern lineage [15]. IL-10 is produced and effective at very low concentrations. To test whether the differential inflammatory response observed in T1-MDMs might be influenced by variations in production and consumption of IL-10 that were not detected by ELISA, we repeated infection experiments in the presence of anti-IL-10 blocking antibodies. Consistent with the idea that IL-10 is produced and acting at very low levels, IL-10 blockage resulted in a systematic increase in pro-inflammatory cytokines TNFα and IL-6, but not in IL-12p40/p70. However, it did not affect the differential between ancient and modern lineages (Figure 8).

**Figure 9:**
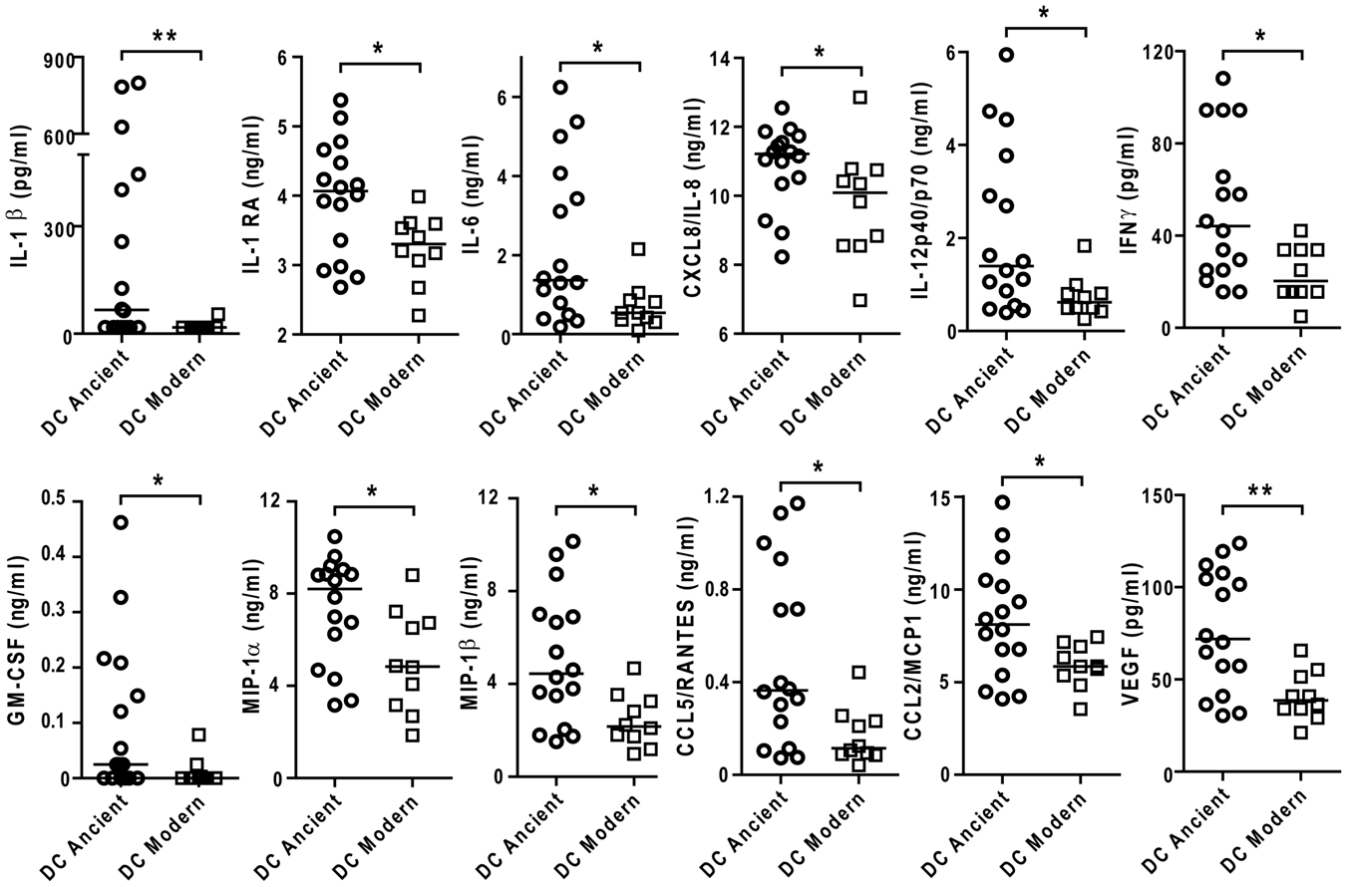
The difference in inflammatory response to ancient and modern lineages is also observed in monocyte-derived DCs although the pattern of secreted cytokines/chemokines is partially different. Scatter plot representation of monocyte derived dendritic cell cytokine responses towards ancient versus modern groups of strains. Filtered supernatants of MD-DCs 24 h post-infection from three independent donors were pooled, analyzed using multiplex technology and resulting values were plotted for each individual cytokine/chemokine. Results show a significant trend towards higher production of each cytokine or chemokine from MD-DCs infected by strains belonging to the ancient lineages (Mann-Whitney U test, * *P*<0.05, **P<0.01).

We also matured monocytes from three different donors in the presence of GM-CSF and IL-4 in order to generate monocyte-derived dendritic cells (MD-DCs) [21]. Compared with autologous T1-MDMs, MD-DCs significantly down-regulated CD14 and expressed higher levels of MHC class II molecules and CD86 (Figure S4). The response of MD-DCs to infection with the different strains resembled that of autologous T1-MDMs. Although the absolute amounts of immune mediators were generally lower, the trend towards weaker responses to the modern lineage was preserved for a largely overlapping panel of cytokines and chemokines but also IL-1β, IL-1RA, CXCL8 and GM-CSF (Figure 9).

## Discussion

Using a panel of strains representative of human MTBC genetic variability, we have found that genetically diverse strains of MTBC vary widely in their induction of an early inflammatory response during infection of human macrophages, and that these differences are linked to MTBC lineages. Overall there was a significantly lower response to evolutionarily modern lineages as compared to ancient lineages.

Previous reports have described differences amongst *M. tuberculosis* isolates in their inflammatory phenotype [10], [12], [13], [15], [18], [19] but the present study is the first to link this to MTBC phylogeny. Consistent with previous reports, we observed a higher inflammatory phenotype for the laboratory strain H37Rv in comparison to the Beijing strain, HN878 [11]. We found that these differences were robust and reproducible using different batches of bacteria to infect monocyte-derived macrophages and autologous dendritic cells across different human donors. We were concerned that an experimental bias could result from inaccuracies in quantifying bacterial preparations. Our quantitation is based on measurement of colony forming units and, while we made every effort to use comparable actively growing cultures, to minimize clumping artifacts and to repeat multiple measurements, errors could arise if there is a variation between strains in the rate of accumulation of dead cells during culture. While it is difficult to rigorously exclude this possibility, we consider it an unlikely explanation for our results since (a) differences in accumulation of dead cells would need to span several orders of magnitude, and (b) in some experimental systems – infection of PBMCs or T2-MDMs – the same preparations showed opposite or no differences in cytokine response. Convergence in responses of T1-MDMs to different strains at later time points is also consistent with equivalence between preparations.

Although we did observe a link between inflammatory phenotype and the various bacterial genotype clusters, each of the phylogenetic lineages included strains that induced high and low levels of inflammatory cytokines. For example, strain N0024 belonging to Lineage 3 consistently elicited a stronger inflammatory response as compared to the other strains from the same lineage. Future comprehensive genetic and biochemical analysis of these strains will be performed with the aim of deciphering the origin of this difference. The variation observed within lineages suggests that differences in inflammatory phenotype cannot be explained simply by the presence or absence of a single molecular determinant, and one model to account for the dispersal of high and low inflammatory strains across the phylogenetic tree is that a diverse range of mutations might influence innate immune recognition and arise independently in different lineages. This model is consistent with the known complexity and diversity of mycobacterial products that have been shown to stimulate or inhibit inflammatory responses [11], [25], [26], [27], [28]. Considering the multiple families of cell-surface and intracellular receptors involved in mycobacterial recognition [29], one might expect that the consequences of molecular changes that affect different ligands would depend on the exact receptor repertoire expressed by different donors [20]. However, the consistent hierarchy in inflammatory response that was observed across independent donors suggests that there is limited human variability in the initial immune response to genetically different mycobacteria. Our demonstration of a robust hierarchy in inflammatory phenotype within the different lineages of MTBC poses a challenge to our understanding at a molecular level of the microbiology and pathogenesis of tuberculosis as multiple mechanisms might converge towards similar phenotypic effects in distinct MTBC lineages. This observation may hold important lessons for development of new vaccines.

In spite of the heterogeneity within MTBC lineages, we observed a statistically significant distribution towards more pro-inflammatory strains amongst members of the ancient lineages, and lower inflammatory responses to strains from the modern lineages. The low inflammatory phenotype of modern strains is in agreement with previous studies of individual Beijing strains [10], [12] and other strains [15] belonging to the modern lineages. Our results are also consistent with the previous suggestion that a low inflammatory response may lead to a reduction in the adaptive response [9].

A possible model to rationalize this finding is that the respective high and low inflammatory responses could reflect different virulence strategies that emerged during the evolution of the ancient and modern lineages. The characteristic latency in TB has been suggested to represent an evolutionary adaptation to low host densities, with reactivation after several decades allowing the pathogen to access new susceptible birth cohorts [1], [30]. By contrast, the low inflammatory response induced by evolutionary modern outbreak strains has been associated with an enhanced ability to cause early progressive disease [10], [15]. Such a strategy may be an advantage in the context of high human population densities, where the number of susceptible hosts is large, and rapid lethality does not threaten to exhaust the pool of new uninfected hosts.

We previously presented an evolutionary scenario for human TB based on population genetic analyses of multilocus sequence data [1] referred to recently as “the most well defined phylogeny of the MTB complex” [31]. According to this scenario, *M. tuberculosis* originated in Africa and accompanied early modern humans on their Out-of-Africa migrations. In those times, human populations were small, and *M. tuberculosis* might have benefited from the latency strategy [30]. During the last few hundred years, the three modern lineages of *M. tuberculosis* experienced strong population expansions as a consequence of the recent human population increases in Europe, India and China [1]. The overall lower inflammatory responses observed in the modern lineages of *M. tuberculosis* might be a consequence of their access to rapidly increasing numbers of susceptible hosts resulting in selection for faster progression to active disease. In support of this hypothesis, a study in the Gambia showed that members of the modern strain lineages were three times more likely than members of the ancient lineages to cause active TB in recently exposed contacts [32].

To our knowledge, this is the first time that the immune response to a particular infectious agent has been measured in a systematic manner by selecting representative strains belonging to the major human MTBC lineages and grasping the global *M. tuberculosis* genetic diversity, including notably *M. africanum*. As we show here, the combination of genotypic, phenotypic and epidemiological studies offers the potential for novel insights into the biology of this pathogen.

## Methods

### Mycobacterial cultures and single cell stock preparation

Mycobacterial cultures of clinical isolates were obtained from a single colony forming unit. One volume of a stationary phase culture of mycobacteria in Middlebrook 7H9 medium with ADC supplement (BD Biosciences), 0.05% Tween-80 (Sigma-Aldrich) and in some cases sodium pyruvate 40 mM (e.g. *M. africanum* strains [33]) was diluted with 100 volumes of the same medium in the absence of detergent and incubated for 10 days at 37°C. Gentle culture dispersion was performed manually every 48 h. Mycobacteria were pelleted, supernatants discarded and pellets dispersed by manual shaking for 1 min with equal volumes of 2–3 mm glass beads. Mycobacteria were resuspended in PBS and centrifuged at 260 xg for 10 min to remove clumps. Cleared supernatants mostly composed of single particles [34] were supplemented with 5% glycerol and titrated on 7H11 agar plates complemented with OADC (BD Biosciences) and sodium pyruvate 40 mM before and after freezing and storage.

### Blood processing and isolation of peripheral blood mononuclear cells

Peripheral blood mononuclear cells (PBMCs) from healthy anonymous donors were isolated from buffy coats processed by the National Blood Services, Colindale, UK. PBMCs were prepared on a Ficoll-Paque density gradient (Amersham Biosciences AB, Uppsala, Sweden) by centrifugation (800 xg, 30 min at room temperature). Recovered PBMCs were washed twice with RPMI (Gibco, Invitrogen) and resuspended in RPMI/FCS(4%)/methyl-cellulose(2%)/DMSO(9%) for gradual overnight freezing in a NalgeneTM Cryo 1°C container before storage in liquid nitrogen.

### Monocyte purification, macrophage differentiation, LPS stimulation and infection

Monocytes were selected from fresh or frozen PBMCs by magnetic cell sorting using CD14 microbeads (Miltenyi Biotec, Auburn, CA, USA) according to manufacturer's recommendations. Cell purity checked by flow cytometry was always >95%. Macrophages were differentiated from monocytes after 6 days of culture in the presence of recombinant human GM-CSF or M-CSF (Peprotech Ltd) for T1-MDMs or T2-MDMs respectively and monocyte derived dendritic cells (MD-DCs) in the presence of GM-CSF and IL-4 as previously described [21]. Cells were recovered after 15 min Trypsin/EDTA (2 mM) treatment, resuspended in RPMI and 5% FCS, and evenly distributed at 8×10^4^ to 1×10^5^/well (according to experiment) in tissue culture treated 96 well plates or 12.5×10^3^/well in 384 well plates before mycobacterial infection at a multiplicity of infection of 1∶1 unless specified otherwise. LPS stimulation was performed at a final concentration of 10 ng/ml. IL-10 blocking experiments were performed as described elsewhere [35], antibodies were added prior to infection at a final concentration of 0.1 µg/ml.

### Bone marrow-derived macrophages

Mice were bred in the animal facilities of the National Institute for Medical Research and provided after being sacrificed in line with code of practice for the humane killing of animals under schedule 1 to the animals (scientific procedures) Act 1986. Authors were not involved in the handling and/or sacrificing of live mice. Femurs from dead Balb/c mice were flushed with 1 ml complete medium (RPMI1640, 1 mM sodium pyruvate, 2 mM glutamine, 10 mM HEPES, 0.05 mM β-mercapthoethanol and 10% FCS) using a 25 G needle. Cells were pelleted, red blood cells lysed for 5 mins with 10 ml 0.83% ammonium chloride, filtered through a 70 µM strainer and washed twice with complete medium before incubation in a CO_2_ incubator at 37°C for 4 days in 90 mm Petri dishes (4×10^6^ in 8 ml complete medium containing 20% L-cell medium). On day 4, 10 ml conditioned medium was added and cells were harvested on day 7 by removing supernatant and adding 5 ml PBS containing 2 mM EDTA to detach the macrophages. Recovered cells were pelleted and resuspended in complete medium and plated out as described for human monocyte derived macrophages.

### ELISA and multiplex analysis

Cell supernatants were recovered at indicated time points, sterilised twice using 96 well filter plates (0.2 µm, Corning) and stored at −20°C until analysis. IL-6, IL-12p40/p70, TNF-α and IL-10 were measured using either ELISA kits (Peprotech) or Luminex 30-plex kit (Invitrogen) following manufacturer's recommendations.

### Statistical analysis

Data analysis, correlation study, paired t-tests, Mann-Whitney U tests and Kruskal-Wallis rank test were performed using GraphPad Prism software and STATA s.e.m. version 10. Without assuming a pre-defined distribution of the response tested, non-parametric statistical analysis has been used all across the study. Unless otherwise stated, we used the individual measures for each combination of donor and strain for all the statistical analysis (n = 200). Principal component analysis was conducted using STATA s.e.m. version 10 with the polymorphic positions found in Hershberg et al. 2008 for the strains used in the present study.

## Supporting Information

Figure S1 Strain-related hierarchy in inflammatory response is conserved between human and murine macrophages. GM-CSF human monocyte derived macrophages (T1-MDMs) and bone-marrow derived macrophages (BMDMs) from Balb-C mice were simultaneously infected with the panel of MTBC strains for 24 h, MOI 1∶1. A) Supernatants were analyzed for IL-6 content showing a very similar pattern in the cytokine response towards each individual strain although at a lower scale for BMDMs. B) Data clustering revealed significantly higher levels of pro-inflammatory cytokine induction by the ancient lineages in both T1-MDMs and BMDMs (Mann-Whitney U test, * *P*<0.05).
(0.53 MB TIF)

Figure S2 Comparison of cytokine profiles from T1-MDMS, T2-MDMs, Mo-DCs and PBMCs according to the three main phylogenetic clusters resulting from PCA analysis. Detailed statistical analysis according to the three phylogenetic cluster resulting from the Principal Component Analysis of Figure 5 (panel A), Figure 6 (panel B), Figure 7A (panel C), Figure 7C-E (panel D) and Figure 9 (panel E).
(1.35 MB TIF)

Figure S3 Differential responses to ancient and modern lineages are consistent across mycobacterial preparations. A second set of bacterial suspensions was prepared and macrophage response tested. A) Scatter plot representation of IL-6 production of human monocyte derived macrophages induced by the different strains of MTBC using two different bacterial preparations. (Experiments were performed independently on two different donors) Spearman test results show significant correlation between batches. B) Differential inflammatory response between ancient and modern lineage was reproduced using an independent preparation of bacteria (Mann-Whitney U test, * *P*<0.05).
(0.20 MB TIF)

Figure S4 T1-MDMs, T2-MDMs, and MD-DCs flow cytometry analysis. Autologous monocytes were differentiated into T1-MDMs, T2-MDMs, or MD-DCs and checked by flow cytometry for CD14, HLA-DR and CD86 expression. A) Forward Scatter versus Side Scatter signal for each cell population represented as a pseudocolor density plot highlighting gating strategy. Expression for each marker has been represented using histograms overlaying isotype control (red line) with specific antibody staining (green line). B) Table summarising ratios of Mean Fluorescence Intensity (MFI) between isotype control and specific antibody staining. Values represent average MFI of three independent donors +/− standard deviation. MD-DCs down-regulated CD14 and expressed high levels of class II MHC and CD86. T1-MDMs express all three markers at lower levels when compared to T2-MDMs.
(3.23 MB TIF)

## References

[1] Hershberg R, Lipatov M, Small PM, Sheffer H, Niemann S (2008). High functional diversity in *Mycobacterium tuberculosis* driven by genetic drift and human demography.. PLoS Biol.

[2] Comas I, Chakravartti J, Small PM, Galagan J, Niemann S (2010). Human T cell epitopes of *Mycobacterium tuberculosis* are evolutionarily hyperconserved.. Nat Genet.

[3] de Jong BC, Antonio M, Gagneux S (2010). *Mycobacterium africanum* review of an important cause of human tuberculosis in West Africa.. PLoS Negl Trop Dis.

[4] Gagneux S, Small PM (2007). Global phylogeography of *Mycobacterium tuberculosis* and implications for tuberculosis product development.. Lancet Infect Dis.

[5] Nicol MP, Wilkinson RJ (2008). The clinical consequences of strain diversity in *Mycobacterium tuberculosis*.. Trans R Soc Trop Med Hyg.

[6] Coscolla M, Gagneux S (2010). Does M. tuberculosis genomic diversity explain disease diversity?. Drug Discov Today: Dis Mech.

[7] Caws M, Thwaites G, Dunstan S, Hawn TR, Lan NT (2008). The influence of host and bacterial genotype on the development of disseminated disease with *Mycobacterium tuberculosis*.. PLoS Pathog.

[8] Kong Y, Cave MD, Zhang L, Foxman B, Marrs CF (2007). Association between *Mycobacterium tuberculosis* Beijing/W lineage strain infection and extrathoracic tuberculosis: Insights from epidemiologic and clinical characterization of the three principal genetic groups of *M. tuberculosis* clinical isolates.. J Clin Microbiol.

[9] Rakotosamimanana N, Raharimanga V, Andriamandimby SF, Soares JL, Doherty TM (2010). Variation in IFN-{gamma} responses to different infecting strains of *Mycobacterium tuberculosis* in AFB smear positive patients and household contacts in Antananarivo, Madagascar.. Clin Vaccine Immunol.

[10] Manca C, Reed MB, Freeman S, Mathema B, Kreiswirth B (2004). Differential monocyte activation underlies strain-specific *Mycobacterium tuberculosis* pathogenesis.. Infect Immun.

[11] Reed MB, Domenech P, Manca C, Su H, Barczak AK (2004). A glycolipid of hypervirulent tuberculosis strains that inhibits the innate immune response.. Nature.

[12] Tanveer M, Hasan Z, Kanji A, Hussain R, Hasan R (2009). Reduced TNF-alpha and IFN-gamma responses to Central Asian strain 1 and Beijing isolates of *Mycobacterium tuberculosis* in comparison with H37Rv strain.. Trans R Soc Trop Med Hyg.

[13] Lopez B, Aguilar D, Orozco H, Burger M, Espitia C (2003). A marked difference in pathogenesis and immune response induced by different *Mycobacterium tuberculosis* genotypes.. Clin Exp Immunol.

[14] Hanekom M, van der Spuy GD, Streicher E, Ndabambi SL, McEvoy CR (2007). A recently evolved sublineage of the *Mycobacterium tuberculosis* Beijing strain family is associated with an increased ability to spread and cause disease.. J Clin Microbiol.

[15] Newton SM, Smith RJ, Wilkinson KA, Nicol MP, Garton NJ (2006). A deletion defining a common Asian lineage of *Mycobacterium tuberculosis* associates with immune subversion.. Proc Natl Acad Sci U S A.

[16] Sinsimer D, Huet G, Manca C, Tsenova L, Koo MS (2008). The phenolic glycolipid of *Mycobacterium tuberculosis* differentially modulates the early host cytokine response but does not in itself confer hypervirulence.. Infect Immun.

[17] Marquina-Castillo B, Garcia-Garcia L, Ponce-de-Leon A, Jimenez-Corona ME, Bobadilla-Del Valle M (2009). Virulence, immunopathology and transmissibility of selected strains of *Mycobacterium tuberculosis* in a murine model.. Immunology.

[18] Hoal-van Helden EG, Stanton LA, Warren R, Richardson M, van Helden PD (2001). Diversity of in vitro cytokine responses by human macrophages to infection by *Mycobacterium tuberculosis* strains.. Cell Biol Int.

[19] Theus SA, Cave MD, Eisenach KD (2005). Intracellular macrophage growth rates and cytokine profiles of *Mycobacterium tuberculosis* strains with different transmission dynamics.. J Infect Dis.

[20] Gagneux S, DeRiemer K, Van T, Kato-Maeda M, de Jong BC (2006). Variable host-pathogen compatibility in *Mycobacterium tuberculosis*.. Proc Natl Acad Sci U S A.

[21] Verreck FA, de Boer T, Langenberg DM, Hoeve MA, Kramer M (2004). Human IL-23-producing type 1 macrophages promote but IL-10-producing type 2 macrophages subvert immunity to (myco)bacteria.. Proc Natl Acad Sci U S A.

[22] Brosch R, Gordon SV, Marmiesse M, Brodin P, Buchrieser C (2002). A new evolutionary scenario for the *Mycobacterium tuberculosis* complex.. Proc Natl Acad Sci U S A.

[23] de Waal Malefyt R, Abrams J, Bennett B, Figdor CG, de Vries JE (1991). Interleukin 10(IL-10) inhibits cytokine synthesis by human monocytes: an autoregulatory role of IL-10 produced by monocytes.. J Exp Med.

[24] Saraiva M, O'Garra A (2010). The regulation of IL-10 production by immune cells.. Nat Rev Immunol.

[25] Ishikawa E, Ishikawa T, Morita YS, Toyonaga K, Yamada H (2009). Direct recognition of the mycobacterial glycolipid, trehalose dimycolate, by C-type lectin Mincle.. J Exp Med.

[26] Stewart GR, Wilkinson KA, Newton SM, Sullivan SM, Neyrolles O (2005). Effect of deletion or overexpression of the 19-kilodalton lipoprotein Rv3763 on the innate response to *Mycobacterium tuberculosis*.. Infect Immun.

[27] Nigou J, Vasselon T, Ray A, Constant P, Gilleron M (2008). Mannan chain length controls lipoglycans signaling via and binding to TLR2.. J Immunol.

[28] Rao V, Fujiwara N, Porcelli SA, Glickman MS (2005). *Mycobacterium tuberculosis* controls host innate immune activation through cyclopropane modification of a glycolipid effector molecule.. J Exp Med.

[29] Jo EK (2008). Mycobacterial interaction with innate receptors: TLRs, C-type lectins, and NLRs.. Curr Opin Infect Dis.

[30] Blaser MJ, Kirschner D (2007). The equilibria that allow bacterial persistence in human hosts.. Nature.

[31] Smith NH, Hewinson RG, Kremer K, Brosch R, Gordon SV (2009). Myths and misconceptions: the origin and evolution of *Mycobacterium tuberculosis*.. Nat Rev Microbiol.

[32] de Jong BC, Hill PC, Aiken A, Awine T, Antonio M (2008). Progression to active tuberculosis, but not transmission, varies by *Mycobacterium tuberculosis* lineage in The Gambia.. J Infect Dis.

[33] Keating LA, Wheeler PR, Mansoor H, Inwald JK, Dale J (2005). The pyruvate requirement of some members of the *Mycobacterium tuberculosis* complex is due to an inactive pyruvate kinase: implications for in vivo growth.. Mol Microbiol.

[34] N'Diaye EN, Darzacq X, Astarie-Dequeker C, Daffe M, Calafat J (1998). Fusion of azurophil granules with phagosomes and activation of the tyrosine kinase Hck are specifically inhibited during phagocytosis of mycobacteria by human neutrophils.. J Immunol.

[35] Conti L, Cardone M, Varano B, Puddu P, Belardelli F (2008). Role of the cytokine environment and cytokine receptor expression on the generation of functionally distinct dendritic cells from human monocytes.. Eur J Immunol.

