# Supplementary figures and images for: Human Macrophage Responses to Clinical Isolates from the Mycobacterium tuberculosis Complex Discriminate between Ancient and Modern Lineages
Source: PLoS Pathog. 2011 Mar 3;7(3):e1001307. doi: 10.1371/journal.ppat.1001307 (PMC3048359; doi:10.1371/journal.ppat.1001307)

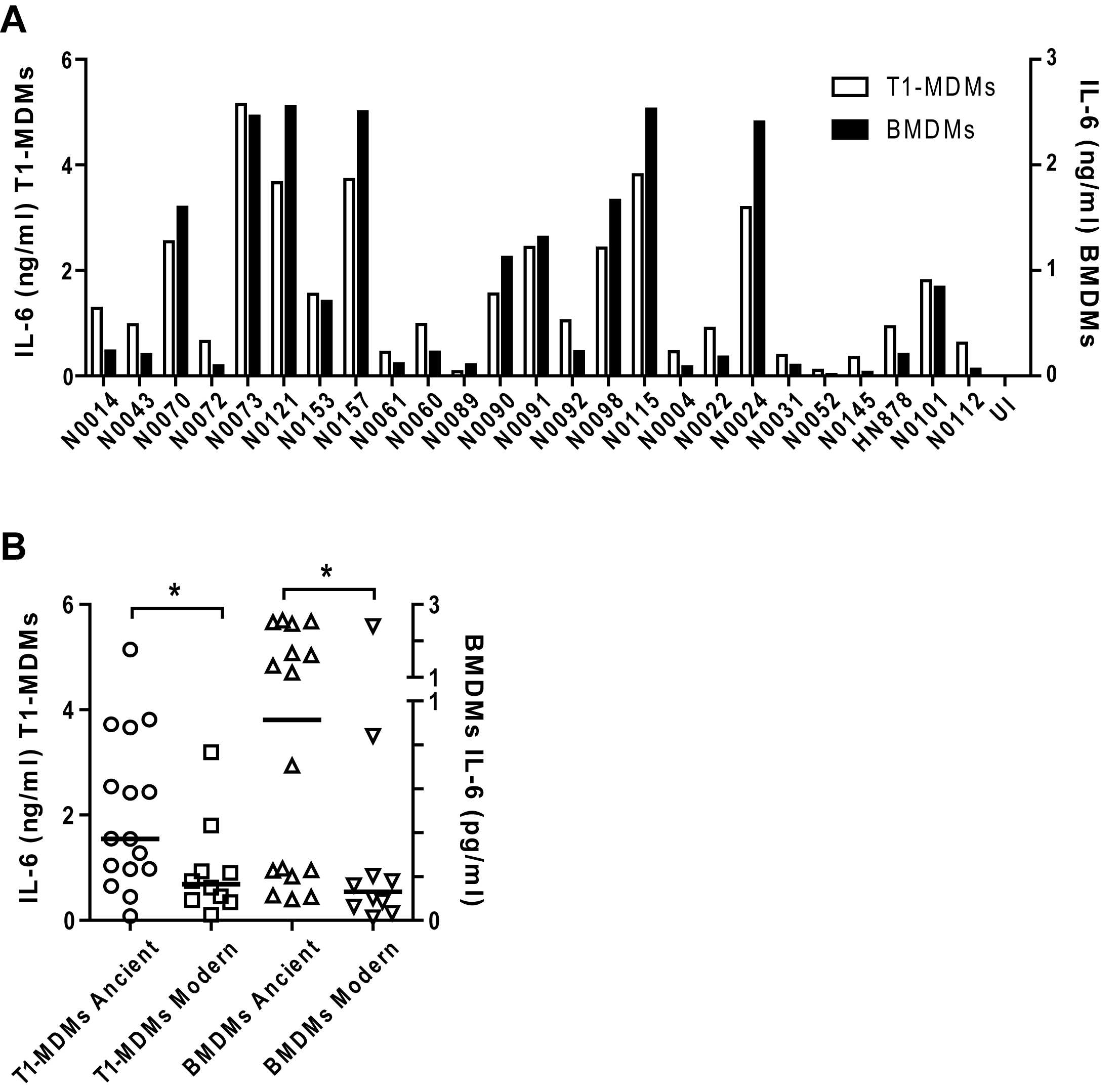

Supplement: Figure S1 — Strain-related hierarchy in inflammatory response is conserved between human and murine macrophages. GM-CSF human monocyte derived macrophages (T1-MDMs) and bone-marrow derived macrophages (BMDMs) from Balb-C mice were simultaneously infected with the panel of MTBC strains for 24 h, MOI 1∶1. A) Supernatants were analyzed for IL-6 content showing a very similar pattern in the cytokine response towards each individual strain although at a lower scale for BMDMs. B) Data clustering revealed significantly higher levels of pro-inflammatory cytokine induction by the ancient lineages in both T1-MDMs and BMDMs (Mann-Whitney U test, * P<0.05). (0.53 MB TIF) [file ppat.1001307.s001.tif]

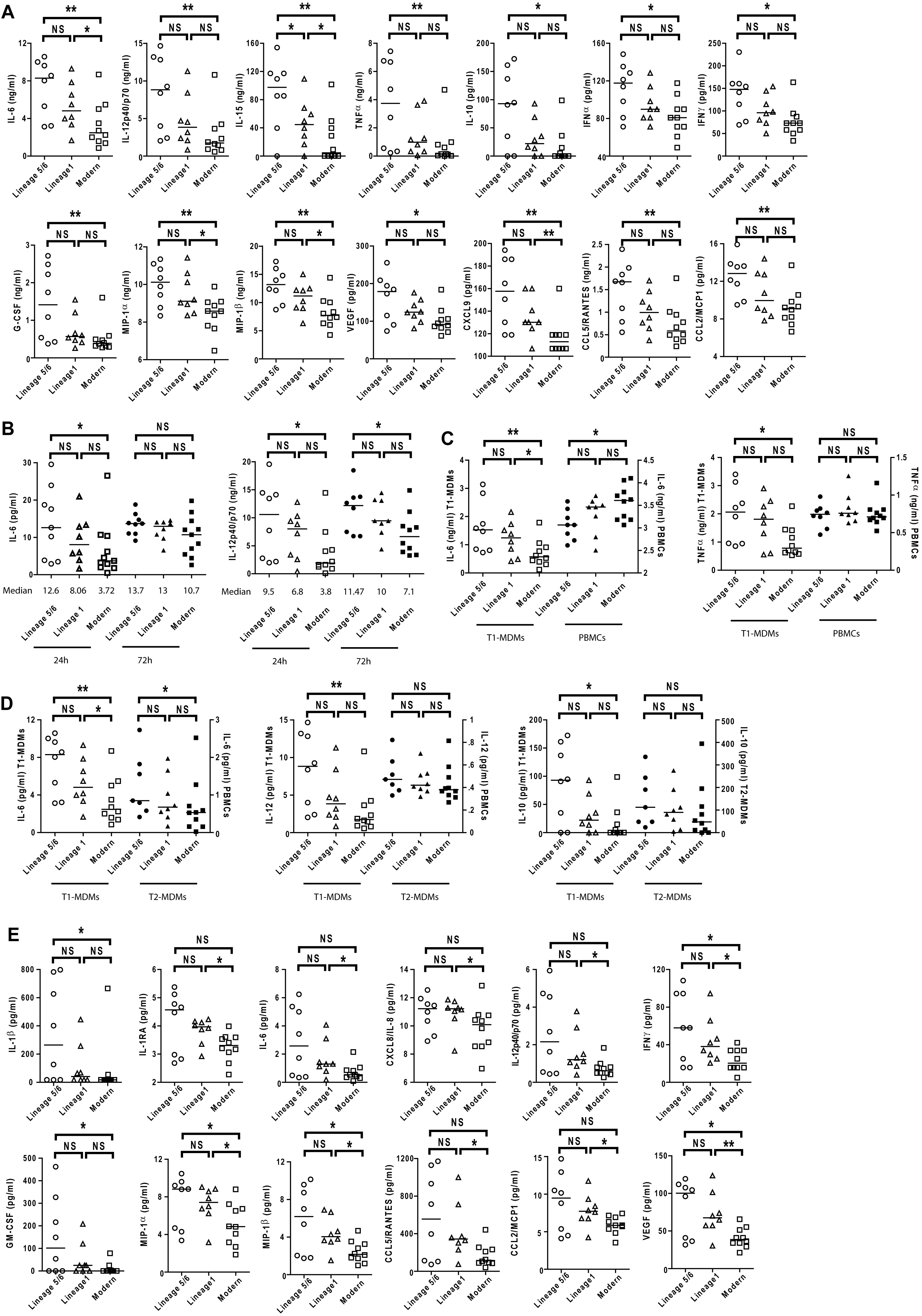

Supplement: Figure S2 — Comparison of cytokine profiles from T1-MDMS, T2-MDMs, Mo-DCs and PBMCs according to the three main phylogenetic clusters resulting from PCA analysis. Detailed statistical analysis according to the three phylogenetic cluster resulting from the Principal Component Analysis of Figure 5 (panel A), Figure 6 (panel B), Figure 7A (panel C), Figure 7C-E (panel D) and Figure 9 (panel E). (1.35 MB TIF) [file ppat.1001307.s002.tif]

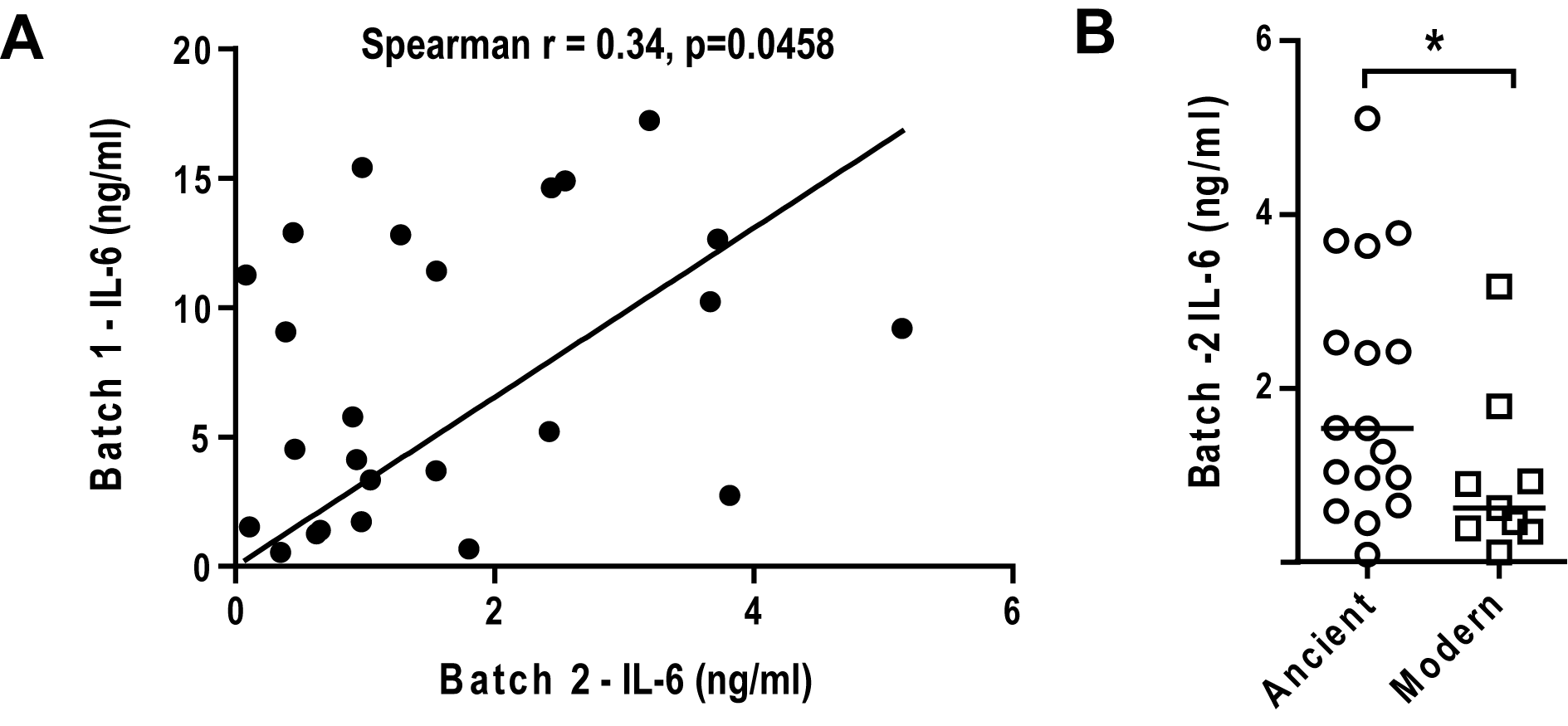

Supplement: Figure S3 — Differential responses to ancient and modern lineages are consistent across mycobacterial preparations. A second set of bacterial suspensions was prepared and macrophage response tested. A) Scatter plot representation of IL-6 production of human monocyte derived macrophages induced by the different strains of MTBC using two different bacterial preparations. (Experiments were performed independently on two different donors) Spearman test results show significant correlation between batches. B) Differential inflammatory response between ancient and modern lineage was reproduced using an independent preparation of bacteria (Mann-Whitney U test, * P<0.05). (0.20 MB TIF) [file ppat.1001307.s003.tif]

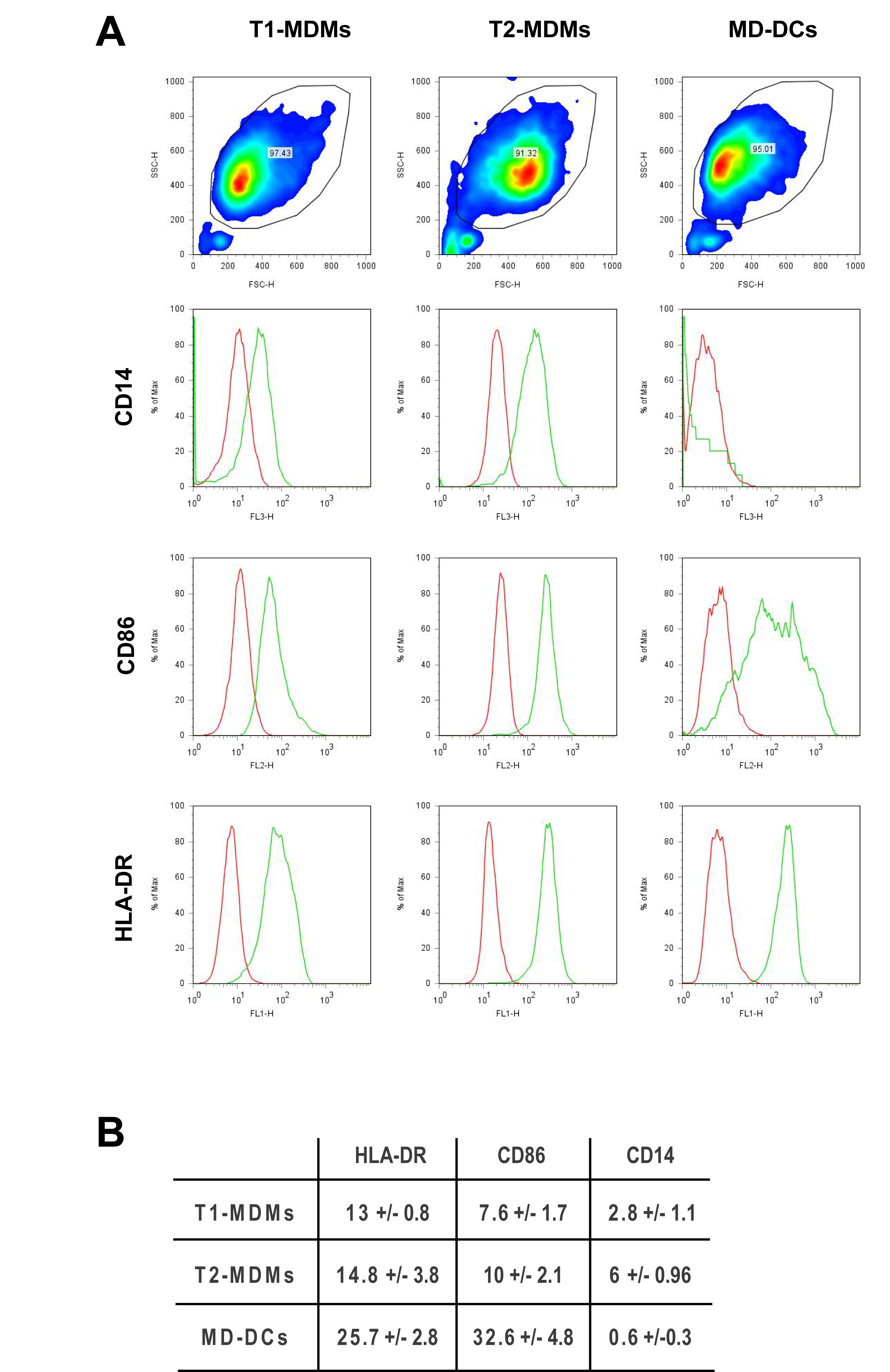

Supplement: Figure S4 — T1-MDMs, T2-MDMs, and MD-DCs flow cytometry analysis. Autologous monocytes were differentiated into T1-MDMs, T2-MDMs, or MD-DCs and checked by flow cytometry for CD14, HLA-DR and CD86 expression. A) Forward Scatter versus Side Scatter signal for each cell population represented as a pseudocolor density plot highlighting gating strategy. Expression for each marker has been represented using histograms overlaying isotype control (red line) with specific antibody staining (green line). B) Table summarising ratios of Mean Fluorescence Intensity (MFI) between isotype control and specific antibody staining. Values represent average MFI of three independent donors +/− standard deviation. MD-DCs down-regulated CD14 and expressed high levels of class II MHC and CD86. T1-MDMs express all three markers at lower levels when compared to T2-MDMs. (3.23 MB TIF) [file ppat.1001307.s004.tif]
